# Absolute Configuration Assignment to Chiral Natural Products by Biphenyl Chiroptical Probes: The Case of the Phytotoxins Colletochlorin A and Agropyrenol

**DOI:** 10.1021/acs.jnatprod.9b01068

**Published:** 2020-02-24

**Authors:** Ernesto Santoro, Stefania Vergura, Patrizia Scafato, Sandra Belviso, Marco Masi, Antonio Evidente, Stefano Superchi

**Affiliations:** † Department of Sciences, University of Basilicata, Viale dell’Ateneo Lucano 10, 85100 Potenza, Italy; ‡ Department of Chemical Sciences, University of Naples Federico II, Complesso Universitario Monte San’Angelo, Via Cintia 4, 80126 Napoli, Italy

## Abstract

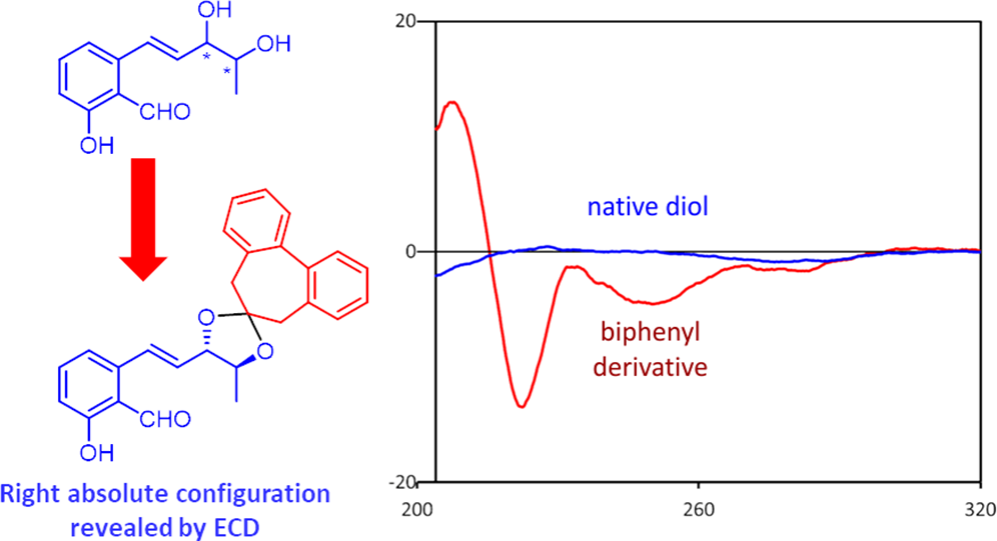

The application of flexible biphenyls as chiroptical probes for the absolute configuration assignment to chiral natural products is described. The method is straightforward and reliable and can be applied to conformationally mobile and ECD silent compounds, not treatable by computational analysis of chiroptical data. By this approach, the (6′*R*) absolute configuration of the phytotoxin colletochlorin A (**1**) was confirmed, while the absolute configuration of the phytotoxin agropyrenol (**2**), previously assigned by the NMR Mosher method, was revised and assigned as (3′*S*,4′*S*). Moreover, with the biphenyl method the configurational assignment can be obtained simply by the sign of a diagnostic Cotton effect at 250 nm in the ECD spectrum, thus allowing application without the need of advanced knowledge of chiroptical spectroscopy and computational protocols.

The assignment of the absolute configuration (AC) is a primary task to be addressed when dealing with chiral molecules and then a relevant issue for medicinal, material, and natural products chemistry. In particular, the key role of absolute configuration on bioactivity of chiral compounds is well-known for naturally occurring metabolites^1,2^ and clearly established for those originating from plants or fungi,^3^ for drugs,^4^ flavors, and fragrances.^5^ Therefore, for the structural characterization of chiral natural products, the assignment of their AC is mandatory, in particular when the biological activity of the compounds has to be investigated. To this end, the classical chemical correlation approaches are very time-consuming, while X-ray diffraction^6^ is not of general applicability.^7^ In fact, often natural products are available in small amounts and/or in noncrystalline form and lack heavy atoms, features that prevent a direct assignment of AC by X-ray diffraction data analysis.^8^ For these compounds the spectroscopic methods based on NMR and chiroptical spectroscopy provide powerful tools for the AC assignment of molecules in solution. The NMR methods are widely applied on natural products,^9,10^ but are essentially semiempirical. Moreover, the most employed one, i.e., the Mosher approach,^11^ is essentially restricted to the assignment of carbinol stereocenters. On the contrary, chiroptical spectroscopies,^12^ such as optical rotation (OR) and optical rotatory dispersion (ORD), electronic circular dichroism (ECD), and vibrational circular dichroism (VCD), provide general, nonempirical, and reliable approaches for AC assignment to natural products. Moreover, ECD spectroscopy also often permits assignment on a microscale using dilute solutions.^13^ In this field a real breakthrough has been achieved in the past two decades, with the advance in the *ab initio* predictions of chiroptical properties. This significantly increased the use of computational methodologies in structural analysis,^14,15^ thus making this approach the method of choice for reliable AC assignments in solution.^16^ However, despite the hundreds of successful applications reported,^17,18^ this approach still remains difficult to apply for highly flexible molecules, displaying many similarly populated conformers and/or giving rise to low chiroptical responses, a common situation encountered in complex natural products. In these cases long and time-consuming conformational analyses are required and the combined use of more than a single chiroptical property is often mandatory to obtain reliable results,^18a,19^ further increasing the analysis complexity. For these difficult cases the use of the so-called “chiroptical probes” offers a practical and reliable alternative, also available to nonspecialists in spectroscopy and computations. Chiroptical probes are achiral moieties which, when linked by either covalent or supramolecular interactions with a chiral nonracemic substrate, give rise to diagnostic signal(s) in the chiroptical spectrum from the sign of which the AC of the investigated molecule can be determined. Such probes are usually chromophoric systems and the chiral induction is revealed by the ECD spectra.^20^ Ideally, chiroptical probes should be molecular systems which simultaneously introduce both rigidity elements in flexible molecules, thus reducing their conformational mobility, and chromophores, thus enhancing the chiroptical response in the ECD spectrum. Unlike the computational methods, this approach has the advantage that a precise spectral prediction is not required, and often a simple visual inspection of the spectrum is enough for the configurational assignment.^21^ Many examples of application of chiroptical probes to AC assignment have been reported, but in all cases the application of the methodology on model compounds of known configuration is reported, and only in a very few examples^22^ such approach is applied to “real life” compounds and natural products of unknown AC.

Some years ago we introduced the use of the 2,2′-bridged biphenyl chiroptical probes, which proved to be versatile, reliable, and efficient tools for AC assignment to chiral diols,^23^ carboxylic acids,^24^ and primary amines.^25^ In these systems, due to the low aryl–aryl rotational barrier displayed by the biphenyl moiety,^26^ a central-to-axial chirality induction occurs between the chiral substrate and the biphenyl itself which, in turn, assumes a preferred *M* or *P* twist depending on the AC of the substrate. Moreover, the sense of the biphenyl twist is readily revealed by the sign of the ECD Cotton effect at 250 nm, in correspondence to the biphenyl A band absorption.^27^ A positive sign of such Cotton effect corresponds to *M* torsion; reversely, a *P* torsion is allied to a negative A band in the ECD spectrum.^28^ Therefore, once the mechanism of twist induction in the biphenyl moiety is determined, the AC of the chiral substrate can be assigned by simply considering the ECD spectrum of the biphenyl derivative. Another relevant advantage of the biphenyl probe is that, unlike most of the reported chiroptical probes, covalently bonded derivatives are obtained. This allows defining the conformations and the substrate-probe interactions more reliably, resorting to both experimental (NMR, X-ray) and computational methods, thus arriving at a more rigorous determination of the twist induction mechanism.

In this paper we will show that the biphenyl chiroptical probe method can be a useful tool for the AC assignment to complex, flexible natural products allowing the configurational assignment in a simple, reliable, and straightforward manner and making the assignment available also to nonspecialists in spectroscopy and computations.

To show this we chose to apply the method to two recently isolated flexible phytotoxins, colletochlorin A (**1**)^29^ and agropyrenol (**2**)^30^ (Figure 1). These compounds were chosen because of the extreme difficulty to be treated by the aforementioned computational approaches. In fact, in both compounds the stereogenic centers are constituted by nonchromophoric chiral diol moieties, thus making them essentially ECD-silent, and which are located on a flexible side chain, so that any computational conformational analysis is quite complex or even unfeasible. On the contrary, the low chiroptical response of both compounds (vide infra) guarantees no significant interference with the diagnostic ECD signals of the biphenyl probe.

**Figure 1:**
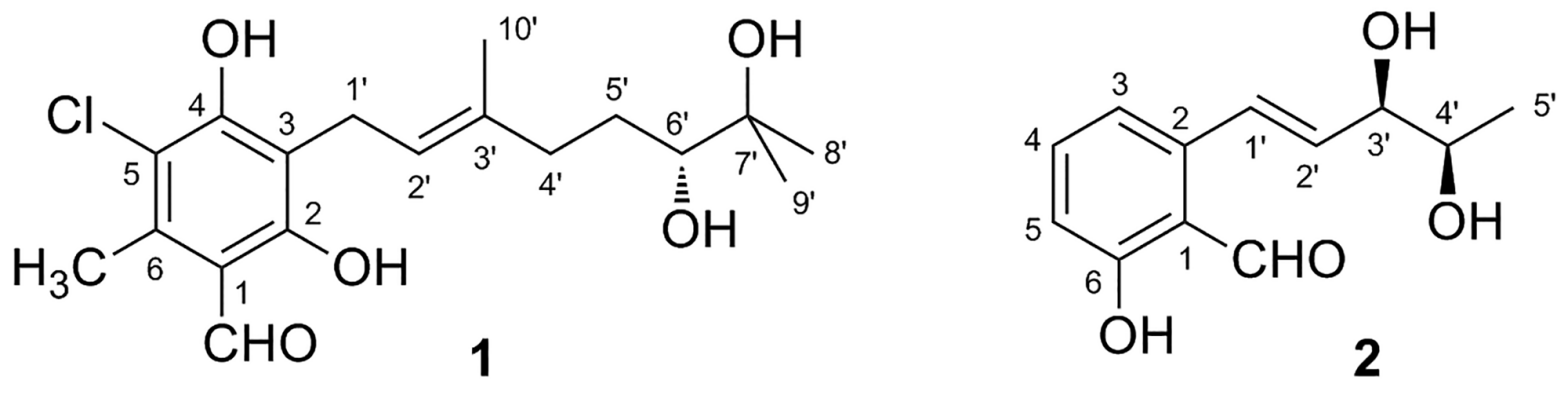
Structures and assigned AC of colletochlorin A (**1**) and agropyrenol (**2**).

## Results and Discussion

### Biphenyl Chiroptical Probe for AC Assignment of Diols

**Scheme 1:**
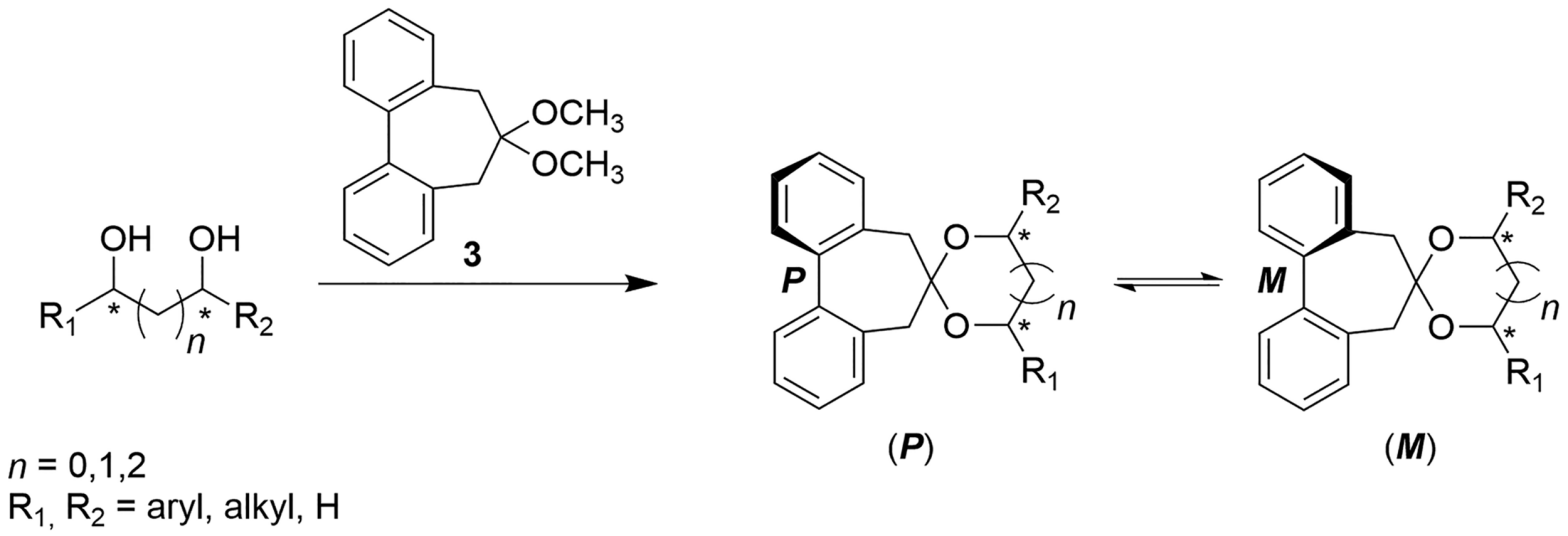
Preparation of Biphenyldioxolanes from Diols

Chiral diols and polyols are extremely common among natural products; the most immediate examples are carbohydrates, but also many natural products isolated from plants, fungi, terrestrial and marine organisms,^31^ possess di- and polyhydroxylated moieties.^32,33^ Therefore, several empirical and nonempirical chiroptical approaches have been developed for their AC assignment. The former are mainly based on the ECD data analysis of diol metal complexes,^34^ while the latter are based on the exciton chirality method (i.e., the Harada–Nakanishi dibenzoate method),^35,36^ or quantum mechanical computations of chiroptical data.^37^ The exciton chirality method requires the presence of two or more chromophoric moieties in the molecules and the knowledge of their spatial arrangement; therefore, a detailed conformational analysis of the substrate is usually required. The same problem arises in the computational approaches, which require the determination of the exact conformer distribution. As a consequence, difficult problems are encountered with conformationally mobile acyclic diols, where the AC assignment is often difficult and troublesome.^36^ A further difficult problem arises with diols devoid of chromophoric moieties in close proximity to the chiral center, that are essentially ECD silent. Several studies have addressed the problem of the use of ECD spectroscopy for AC determination to acyclic diols, showing that the transformation of these compounds in cyclic, conformationally constrained derivatives can provide a practical answer to the problem of the conformation analysis. According to this strategy, *bis*-chromophoric 1,2-diarylethane-1,2-diols were transformed into conformationally fixed 2,2-dimethyl-1,3-dioxolanes,^38^ while monochromophoric 1-arylethane-1,2-diols were converted in the corresponding 4-biphenylboronates,^39^ thus blocking the conformational mobility and, at the same time, adding the second chromophore required by the exciton coupling treatment. The more challenging aliphatic nonchromophoric diols were also approached by us developing a simple, straightforward, and general method for AC assignment to 1,2-, 1,3-, 1,4-cyclic, and acyclic diols by the use of the aforementioned flexible biphenyl chiroptical probes.^23^ According to this approach, diols are transformed in the corresponding biphenyl dioxolanes (Scheme 1), thus obtaining a pair of diastereoisomers having, respectively, *P* and *M* twist of the biphenyl moiety.

**Figure 2:**
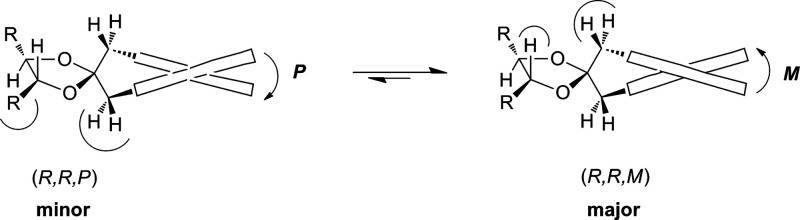
Schematic representation of the conformational equilibrium in biphenyldioxolanes derived from *anti*-1,2-diols.

**Figure 3:**
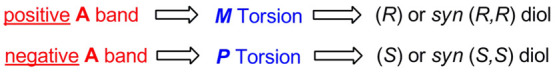
Mnemonic scheme relating AC of *syn* 1,2-, 1,3-diols, and 1,4-diols with sign of the A band in the ECD spectrum of their biphenyldioxolanes.

The low rotational barrier^23^ (ca. 14 kcal/mol) of the biphenyl in these compounds allows, at room temperature, a thermodynamic equilibrium between the two diastereoisomers. Therefore, the most stable is also the major one. The mechanism of chirality induction from the chiral diol to the biphenyl was clarified, revealing that in dioxolanes derived from *syn* (*R,R*) 1,2-, 1,3-, or 1,4-chiral diols,^40^ independently of the diol structure, the most stable diastereoisomer is the one having an *M* torsion of the biphenyl moiety. In fact, from the structures in Figure 2 it can be clearly seen that in the (*R*,*R*,*P*) isomer both the benzylic CH_2_ moieties face a bulky R substituent, while in (*R*,*R*,*M*) the benzylic residues are opposite to hydrogen atoms and then minor steric interactions result. In this system an efficient central-to-axial chirality induction occurs from the diol to the twisted biphenyl; therefore, by simply recognizing the prevailing sense of twist of the biphenyl moiety the AC of the diols can be determined. Moreover, as reported above, the twist of the biphenyl can be readily determined from the ECD spectrum^28^ by the sign of the so-called A band.^41^ Therefore, the simple rule reported in Figure 3 can be proposed, by which, simply looking at the sign of the A band in the ECD spectrum, it is possible to identify the biphenyl torsion and then the AC of the diol inducing the torsion. The same relationship between the sign of the A band, the biphenyl twist, and the diol AC was also demonstrated for mono- and disubstituted and *anti* 1,3- and 1,4-diols.^23^

### AC Assignment to Colletochlorin A (**1**)

The application of the aforementioned method for AC assignment of diols was tested on the naturally occurring phytotoxin colletochlorin A [(+)-**1**], a well-known 3-diprenyl orsellinaldehyde derivative isolated for the first time in 1973 from the culture filtrate of *Colletotrichum nicotianae*, a pathogenic fungus that induces tobacco anthracnose^42^ and more recently from the culture filtrate of *Colletotrichum higginsianum*.^29^ Compound **1** was subsequently isolated also as the main phytotoxin from the culture filtrates of *C. gloeosporioides* and proposed as potential bioherbicide for biocontrol of *Ambrosia artemisiifolia*,^43^ a widespread invasive weed native to North America causing severe crop losses^44^ and huge medical costs as a consequence of its highly allergenic pollen production. The (6′*R*) AC of the carbinol stereocenter of **1** was assigned by applying the advanced Mosher’s method.^29^ Recently, the enantioselective total synthesis of **1** together with that of the closely related colletorin A was carried out by some of us,^45^ which permitted confirmation of its AC. The preparation of some analogues of **1** also allowed comparison of their activity and to show the dependence of herbicidal and insecticidal properties on both the AC and the nature of the halogen substituent.^45^

Notably, neither the computational nor the exciton chirality based chiroptical methods can be used for the AC assignment to **1**. In fact, compound **1** displays a spectrum with low-amplitude Cotton effects (Figure S1, Supporting Information), showing that it is practically ECD silent, and shows a high conformational mobility, displaying more than 2500 populated conformers within a 30 kcal window for a MMFF94 force field molecular mechanics conformational search. Both these features discriminate against AC assignment by the computational analysis of either ECD, VCD, or ORD spectra. In fact, the first requires ECD spectra with relatively high amplitude Cotton effects to be reliably applied and any computational reproduction of chiroptical data needs the determination of structure and relative population of the populated conformers, a task quite difficult to be achieved in the case of a high number of conformers like in **1**. The same problems also make unfeasible the Harada–Nakanishi dibenzoate approach, which requires an accurate conformational analysis as well, to ascertain the actual major dibenzoate conformation.

**Scheme 2:**
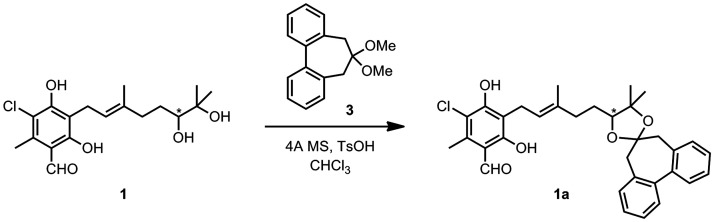
Preparation of the Biphenyldioxolane **1a** from Colletochlorin A (**1**)

**Figure 4:**
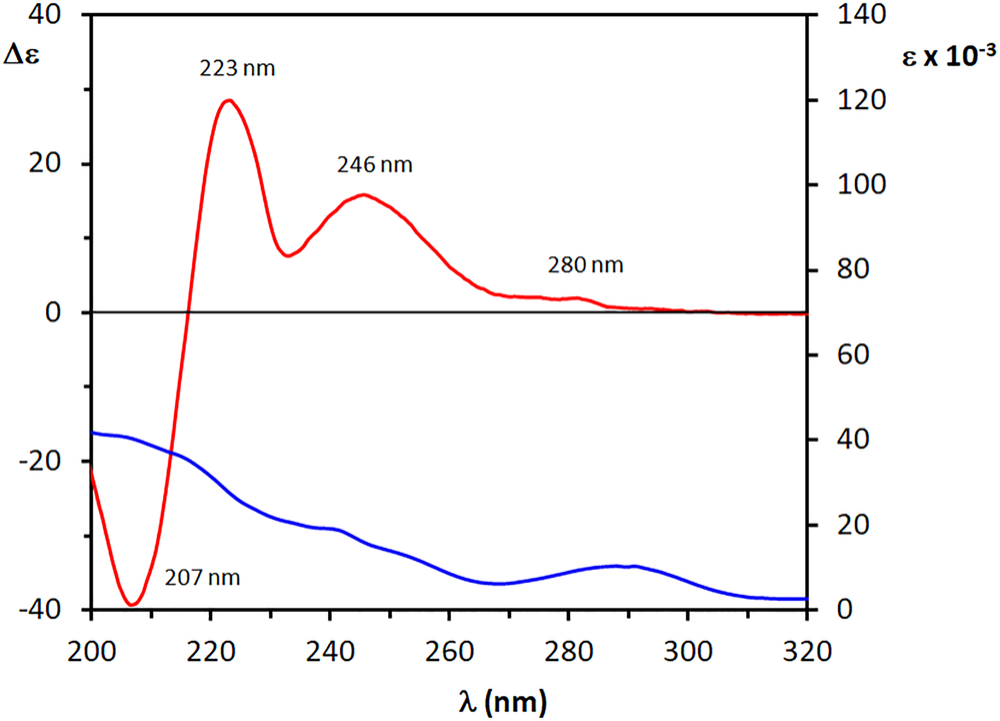
Experimental UV (blue line) and ECD (red line) spectra of **1a** in CH_3_CN.

The flexible biphenyl approach was then applied to (+)-**1** obtained from *C. gloeosporioides* culture^29^ which, following the method described above, was transformed into the corresponding dioxolane **1a** by reaction with dimethylacetal **3** in CHCl_3_, in the presence of traces of *p*-toluene sulfonic acid and 4 Å molecular sieves (Scheme 2). Notably, the derivatization procedure does not require any protection of the phenolic and formyl functionalities, smoothly affording the dioxolane **1a** after filtration, evaporation of the solvent, and chromatographic purification. The ECD and UV spectra of **1a** were recorded (CH_3_CN) in the 200–320 nm range. The UV absorption spectrum of **1a** (Figure 4) shows the typical bands of a biphenyl chromophore with the A band as a shoulder at 242 nm (ε ∼ 18 600) and the more intense absorption at 206 nm (ε ∼ 40 800) of C band,^41^ while the electron transfer π–π* absorption of the benzaldehyde chromophore is visible at 290 nm (ε ∼ 10 200). The ECD spectrum shows high amplitude Cotton effects, indicating the typical features of the biphenyl chromophore as well,^23^ with a positive Cotton effect (Δε = +15.7) occurring at 246 nm (i.e., in correspondence to the A band), followed by a positive couplet-like feature centered at 220 nm with sequential positive and negative Cotton effects at 223 nm (Δε = +28.6) and 207 nm (Δε = −39.3), respectively. A weaker positive Cotton effect is also visible at about 280 nm (Δε = +2.0) in correspondence to the π–π* transition. As reported above, a positive Cotton effect due to the A band is related to an *M* torsion of the C_Ar_–C_Ar_ bond which, according to the rule in Figure 3, permits assignment of (6′*R*) AC to diol (+)-**1**. This AC agrees with the reported data assigned by application of the Mosher’s method,^29^ thus providing an independent confirmation of such empirical assignment.

### AC Assignment to Agropyrenol (**2**)

The same approach was used for the AC assignment to agropyrenol [(−)-**2**], a phytotoxin characterized as a substituted salicylic aldehyde and produced by a strain of *Ascochyta agropyrina* var. *nana*, a fungal pathogen of the perennial weed *Elytrigia repens*, a widespread perennial weed throughout cold temperate regions all over the world.^30^ The reported (3′*R*,4′*R*) AC of **2**(30) was determined by application of the advanced Mosher’s method.^11c^ When assayed on leaves of several weed plants, i.e., *Mercurialis annua*, *Chenopodium album*, and *Setaria viridis*, **2** proved to be phytotoxic, causing the appearance of necrotic lesions, not associated with antibiotic, fungicidal, or zootoxic activity.^30^ Subsequently, a structure–activity relationships (SAR) study was carried out on six semisynthesized derivatives of **2** assaying their phytotoxicity on several weed plants. From the SAR study both the double bond and the diol system of the 3,4-dihydroxypentenyl side chain as well as the C-1 formyl group proved to be important for the phytotoxicity.^47^

The ECD spectrum of (−)-**2** shows Cotton effects with relative higher amplitudes than **1** (Figure S2, SI). In fact, in **2** the stereogenic carbinol moiety is allylic and the adjacent double bond chromophore gives rise to a detectable chiroptical response. Molecular mechanics conformational analysis of **2** with the MMFF94 force field afforded 100 populated conformers which, upon optimization at the DFT/B3LYP/TZVP/gas phase level, provided 15 populated conformers at room temperature (Table S1, SI). It follows that, in principle, **2** could be treated also by computational approaches, even if the weak chiroptical response and the high number of populated conformers could prevent a reliable AC assignment.

**Scheme 3:**
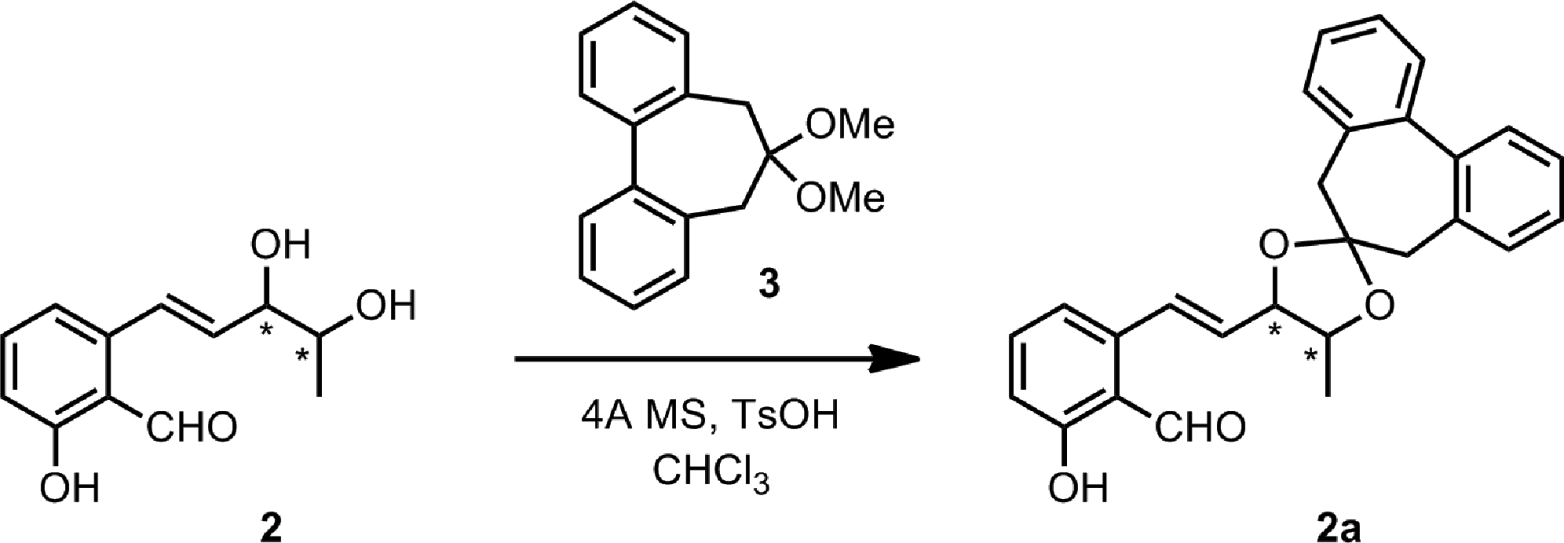
Preparation of Biphenyldioxolane **2a** from Agropyrenol (**2**)

The biphenyl approach was applied to compound **2** by transforming it into the corresponding dioxolane **2a** under the same reaction conditions for **1** (Scheme 3). Again, no protection of both the phenolic and formyl functions was needed, and the dioxolane **2a** was recovered after filtration, evaporation of the solvent, and chromatographic purification.

**Figure 5:**
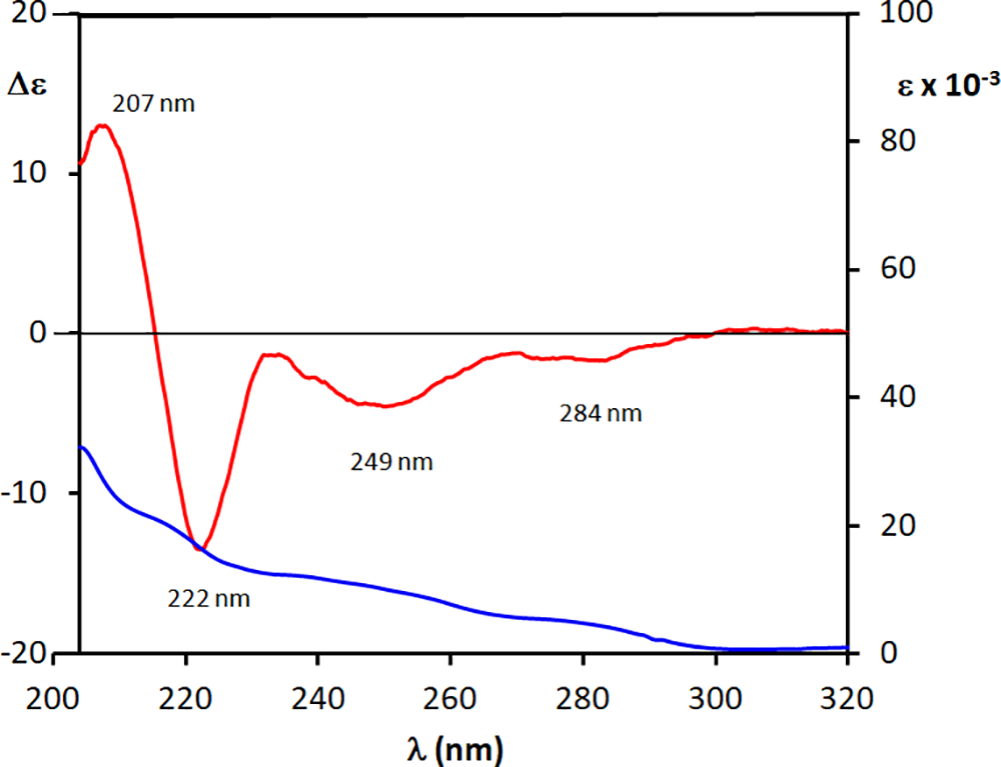
Experimental UV (blue line) and ECD (red line) spectra of **2a** in THF.

The ECD and UV spectra of **2a** were recorded (THF) in the 200–320 nm range. The UV absorption spectrum of **2a** (Figure 5) shows the typical biphenyl bands with the A band as a shoulder at 237 nm (ε ∼ 12 300), the B band as a shoulder at 217 nm (ε ∼ 20 500), and the more intense C band at 205 nm (ε ∼ 32 100), while the π–π* absorption is visible as a weaker band at 280 nm (ε ∼ 4900). The ECD spectrum shows the main features of the biphenyl chromophore,^23^ with the negative Cotton effect allied to the A band occurring at 249 nm (Δε = −4.7) and the sequential negative and positive Cotton effects at 222 nm (Δε = −13.6) and at 207 nm (Δε = +13.5), respectively. A negative low-amplitude Cotton effects is also visible at 284 nm (Δε = −1.8) in correspondence to the π–π* carbonyl absorption. The *syn-*relative configuration of the diol moiety was known from NMR data analysis;^30^ therefore, the rule reported in Figure 3 can be safely applied. In fact, for *syn-*diols such qualitative rule can be directly applied, while *anti-*diols require more complex conformational analysis to predict the preferred biphenyl torsion.^23b^ According to such a rule, the presence of a negative Cotton effect in correspondence to the A band is determined by a *P* twist of the biphenyl probe which, in turn, unambiguously reveals an (3′*S*,4′*S*) AC of the derivatized diol (−)-**2**.

A further, independent confirmation of such assignment was provided by comparison of the experimental ECD spectrum of **2a** with that computed for (3′*S*,4′*S*)-**2a**. Therefore, a conformational analysis of (3′*S*,*4′S*)-**2a** was carried out, first by MM computations and then at the DFT/B3LYP/TZVP/gas phase level of theory, providing four *P* and four *M* twisted populated atropoisomers accounting for 78.6 and 21.6% of the overall population, respectively (Table S2, SI). Notably, DFT computations confirmed the preferred *P* torsion of the biphenyl moiety predicted by the rule in Figure 3. For each set of either *P* or *M* diastereoisomers the ECD spectrum was computed at the TDDFT/CAM-B3LYP/aug-cc-pvdz/gas phase level of theory, taking into account the Boltzmann relative populations of the conformers. As inferred from Figure S3 (SI), the experimental ECD spectrum of **2a** fully agrees with the computed one for the *P*/*M* diastereomeric mixture of the (3′*S*,4′*S*) enantiomer, thus allowing confirmation of such AC for the diol (−)-**2**. The experimental ECD spectrum closely resembles the computed one of (*3′S*,4′*S*,*P*)-**2a**, thus independently confirming the correspondence between the negative sign of the Cotton effect at 250 nm and the *P* biphenyl twist empirically established by Mislow and co-workers in the early sixties.^28a,28b^

**Figure 6:**
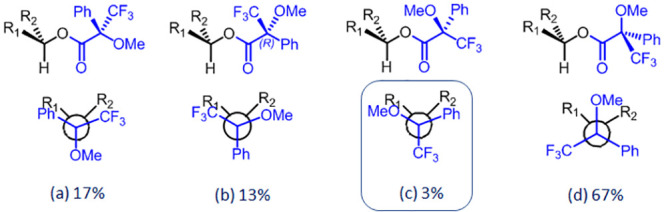
Structures and relative populations of most stable rotamers of the 4′-(*R*)-MTPA ester of (3′*R*,4′*R*)-**2** computed at the DFT/B2LYP/TZVP/IEFPCM/CHCl_3_ level of theory. The structure (c) in the box, accounting for only 3% of the relative populations is the one requested to apply the Mosher’s rule for the AC assignment.

The AC determined in this way is opposite to the reported AC and determined by the NMR Mosher’s method.^30^ Given the wide application of the Mosher’s method, we considered it worthwhile to investigate the reason for its failure in this case. The application of the Mosher's method requires the esterification of the chiral alcohol with both enantiomers of methoxytrifluoromethylphenyl acid (MTPA) and the NMR analysis of the resulting MTPA diastereomeric esters. From the chemical shift differences between the same signals in the (*R*)- and (*S*)-MTPA esters (Δδ), the relative position of the groups linked to the stereogenic carbon and the AC can be established.^10^ However, to apply such method it must be assumed that the most stable conformation of the ester moiety is that represented by structure (c) in Figure 6 in which the hydrogen on the stereogenic center, the ester carbonyl, and the CF_3_ group are eclipsed. It follows that when such conformation is not the major one the correlation between Δδ shift and AC may not be fulfilled. We then carried out a computational conformational analysis on the mono-4′-(*R*)-MTPA ester of compound (3′*R*,4′*R*)-**2**. Such analysis, carried out at the DFT/B3LYP/TZVP/IEFPCM/CHCl_3_ level of theory, provided 10 most populated conformers at room temperature (Table S3, Figure S4, SI), which can be grouped into the four families of rotamers shown in Figure 6. As inferred from Figure 6, in this compound, the rotamer (c), required for the application of the Mosher’s model, accounts for only 3% of the total population, while the most abundant rotamer (d) provides different phenyl shielding effect and, then, an opposite correlation. As shown by the conformer (d) structure in Figure S5 (SI) such conformation can be stabilized by the presence of hydrogen bonding between the free hydroxy group of the diol, the methoxy moiety, and the ester oxygen. This can explain the failure of the Mosher’s assignment and alerts about the uncritical use of such method which, despite its extensive use, remains essentially based on an empirical approach.

In conclusion, we have described herein the first application of flexible biphenyls as chiroptical probes for the AC assignment to chiral natural products. This is also one of the few applications of chiroptical probes to configurational assignments of natural products. The method proved to be particularly straightforward and reliable, allowing confirmation of the (6′*R*) AC of the phytotoxin colletochlorin A [(+)-**1**] and to revise the AC reported for the phytotoxin agropyrenol [(−)-**2**], which has indeed a (3′*S*,4′*S*) AC. We also showed that, in this case, the NMR Mosher’s method, widely employed to assign AC to carbinol stereocenters in natural products, provides a wrong result. The reason for that failure was also clarified by quantum mechanical computations. It must be highlighted that both these phytotoxins were particularly difficult to treat by computational approaches. In fact, both display ORD and ECD spectra with very low intensity and a great conformational flexibility, thus hampering the AC assignment also by VCD spectroscopy, highly sensitive to the distribution of conformers. On the contrary, the use of the biphenyl chiroptical probe provides the correct assignment by simply considering the sign of the diagnostic Cotton effect at 250 nm in the ECD spectrum. Such results show that unlike most of the reported chiroptical probes, the biphenyl probe method can be efficiently and reliably applied even to complex molecules like the natural products and not only to simple model compounds. Moreover, the simplicity of the method allows its application also to nonspecialists in spectroscopy and computations.

## Experimental Section

### General Experimental Procedures

Optical rotations were measured in CHCl_3_ solution on a Jasco DIP-370 digital polarimeter. UV and ECD spectra were recorded at room temperature on a JASCO J815 spectropolarimeter, using 0.1 mm cells and concentrations of about 1 × 10^–3^ M in CH_3_CN and THF solutions. ^1^H NMR spectra were recorded in CDCl_3_ at either 500 or 400 MHz, ^13^C NMR spectra were recorded at 100 MHz in CDCl_3_. GC analyses were performed on a Hewlett-Packard HP 6890 gas chromatograph equipped with a Hewlett-Packard MS 5973 mass selective detector and a fused silica capillary column (HP-5MS; 30 m × 0.25 mm i.d., 0.25 μm film thickness). ESI-MS spectra were recorded on Agilent Technologies 6120 Quadrupole LC-MS instrument. Analytical and preparative TLC were performed on silica gel plates (Merck, Kieselgel 60, F254, 0.25, and 0.5 mm respectively) and column chromatography was performed on silica gel (Merck, Kieselgel 60, 0.063–0.200 mm). The dimethyl acetal **3** was obtained via the corresponding biphenyl-ketone,^48^ by treatment with trimethyl orthoformate, in the presence of *p*-TsOH, followed by neutralization with ammonia dissolved in EtOH.^49^

### Phytotoxin Source

The samples of colletochlorin A (**1**)^29^ and agropyrenol (**2**)^30^ used in this study were obtained from the liquid culture filtrates of *C. gloeosporioides* and *A. agropyrina* var. *nana*, respectively, as previously reported. They were identified comparing their specific rotation, ^1^H NMR, and ESI-MS data with reported data for **1**(29) and for **2**.^30^

### General Procedure for the Synthesis of Biphenyl Dioxolanes

To a solution of the diol (0.36 mmol) in anhydrous CHCl_3_(5 mL) the dimethylacetal **3** (0.36 mmol), 4 Å molecular sieves, and a few crystals of *p*-toluene sulfonic acid were added. The mixture was stirred at rt overnight. After filtration and evaporation of solvent the crude was purified by chromatography on silica gel.

#### Biphenyldioxolane **1a**

Yield 60%; [α]_D_ = +20 (*c* = 0.2, CHCl_3_); ^1^H NMR (500 MHz, CDCl_3_) δ 12.74 (s, 1H, O*H*), 10.10 (s, 1H, C*H*O), 7.42 (t, *J* = 8.6 Hz, 2H), 7.36–7.27 (m, 5H), 7.22 (m, 1H), 7.01 (bs, 1H), 6.39 (m, 1H), 5.34 (m, 1H), 3.72 (m, 1H), 3.46 (m, 2H), 2.81 (m, 1H), 2.65 (m, 1H), 2.54 (m, 4H), 2.24 (m, 1H), 2.13 (m, 1H), 1.83 (s, 3H), 1.63 (m, 1H), 1.53 (m, 1H), 1.26 (s, 3H), 1.19 (s, 3H); ^13^C NMR (100 MHz, CDCl_3_) δ 193.2 (CHO), 162.2, 156.1, 140.1, 137.6, 135.4, 129.6, 129.4, 128.2, 127.6, 127.0, 126.9, 116.0, 114.3, 113.6, 113.1, 107.9, 98.4, 80.1, 67.7, 67.4, 45.2, 43.1, 36.5, 33.2, 29.7, 29.1, 23.9, 23.3, 22.0, 15.9, 14.4. HRESIMS (+) *m*/*z* 569.2075 [M + Na]^+^ (calcd for C_33_H_35_ClNaO_5_ 569.2071).

#### Biphenyldioxolane **2a**

Yield 54%; [α]_D_ = −83 (*c* = 0.2, CHCl_3_); ^1^H NMR (400 MHz, CDCl_3_) δ 11.87 (s, 1H, O*H*); 10.33 (s, 1H, C*H*O); 7.46 (d, *J* = 5.7 Hz, 2H); 7.38 (dt, *J* = 5.7, 1.4 Hz, 2H); 7.32 (d, *J* = 3.2 Hz, 2H); 7.30–7.23 (m, 4H); 6.97 (d, *J* = 6.9 Hz, 1H); 6.91 (d, *J* = 6.9 Hz, 1H); 6.12 (m, 1H), 4.25 (br s, 1H), 4.01 (br s, 1H), 2.85 (d, *J* = 13.4 Hz, 2H); 2.78 (d, *J* = 13.4 Hz, 2H); 1.41 (d, *J* = 6.0, 3H); ^13^C NMR (100 MHz, CDCl_3_) δ 194.96 (CHO), 162.80, 141.59, 140.12, 137.17, 135.61, 129.58, 129.49, 128.41, 128.38, 128.01, 127.65, 127.30, 125.48, 118.93, 118.36, 117.56, 117.22, 44.18, 43.68, 34.20, 31.90, 30.29, 29.67, 22.67, 14.10. HRESIMS (+) *m*/*z* 435.1568 [M + Na]^+^ (calcd for C_27_H_24_NaO_4_ 435.1572).

### Computational Details

Preliminary conformational analyses were performed by the Spartan02 package^50^ employing MMFF94s molecular mechanics force field with Monte Carlo searching and fixing arbitrarily absolute configurations (6′*R*) for **1**, (3′*R*,4′*R*) for **2**, **2a**, and 4′-(*R*)-MTPA-**2**. All possible conformers were searched, considering the degrees of freedom of the system within an energy window of 30 kcal/mol. In the case of (3′*R*,4′*R*)-**2** and **2a** the minimum energy conformers found by molecular mechanics were further fully optimized by using the DFT at the DFT/B3LYP/TZVP level in the gas phase by Gaussian09 package.^51^ The conformations of 4′-(*R*)-MTPA-**2** were fully optimized at the DFT/B3LYP/TZVP level of theory, taking into account the solvent effect by using the IEFPCM implicit model with CHCl_3_ as solvent. All conformers are real minima, no imaginary vibrational frequencies have been found, and the free energy values have been calculated and used to get the Boltzmann population of conformers at 298.15 K. The DFT/B3LYP/TZVP geometries were employed as input geometries for calculation of UV and ECD spectra at the TDDFT/CAM-B3LYP/aug-cc-pVDZ level. The calculated UV and ECD spectra were obtained as average over the conformers Boltzmann populations. The ECD spectra were obtained from calculated excitation energies and rotational strengths, as a sum of Gaussian functions centered at the wavelength of each transition, with a parameter σ (width of the band at 1/2 height) of 0.25 eV. To guarantee origin independence and to evaluate the quality of the molecular wave functions employed, computed ECD spectra were obtained both in the length and velocity representation, using the lowest 30 states. The velocity/length calculated spectra were almost coincident, indicating a good level of calculation. Therefore, in all figures only the velocity-form predicted spectra are reported.

## Supporting Information Available

NMR and ECD spectra and conformational analyses data (PDF)
