# Supplementary material for: Absolute Configuration Assignment to Chiral Natural Products by Biphenyl Chiroptical Probes: The Case of the Phytotoxins Colletochlorin A and Agropyrenol
Source: J Nat Prod. 2020 Feb 24;83(4):1061–8. doi: 10.1021/acs.jnatprod.9b01068 (PMC7997626; doi:10.1021/acs.jnatprod.9b01068)
Supplement: Supplementary file 1 — np9b01068_si_001.pdf [file np9b01068_si_001.pdf]

Supporting Information

Absolute Configuration Assignment to Chiral Natural Products by Biphenyl Chiroptical Probes: the Case of the Phytotoxins Colletochlorin A and Agropyrenol

Ernesto Santoro,[†] Stefania Vergura,[†] Patrizia Scafato,[†] Sandra Belviso,[†] Marco Masi,[‡]

Antonio Evidente,[‡] and Stefano Superchi[†,*]

[†]Department of Sciences, University of Basilicata, Viale dell'Ateneo Lucano 10, 85100 Potenza, Italy.

[‡] Department of Chimcal Sciences, University of Naples Federico II, Complesso Universitario Monte San'Angelo, Via Cintia 4, 80126 Napoli, Italy.

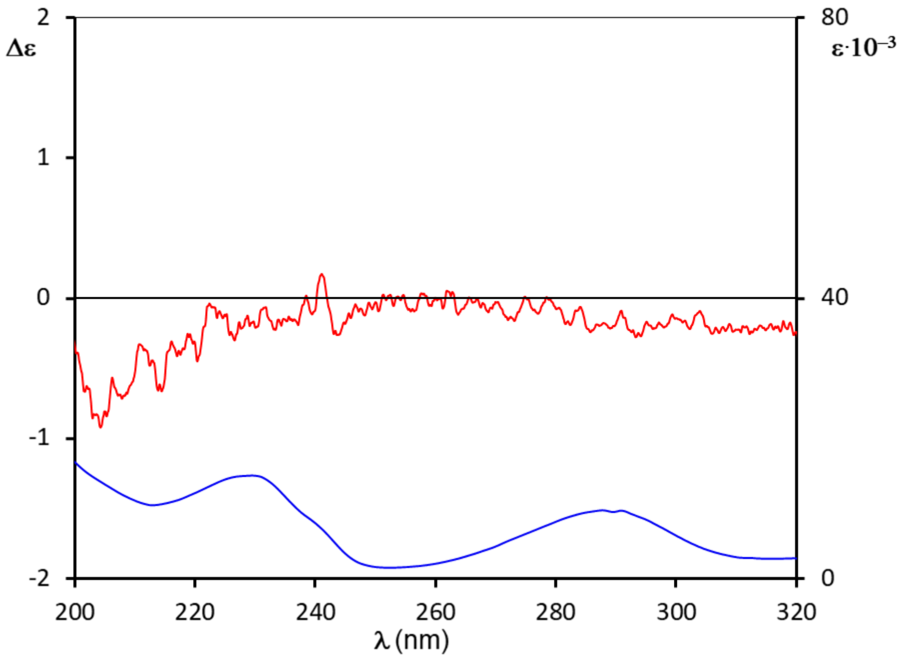

Figure S1. Experimental ECD spectrum of colletochlorin A (-)-**1** in CH_3CN.

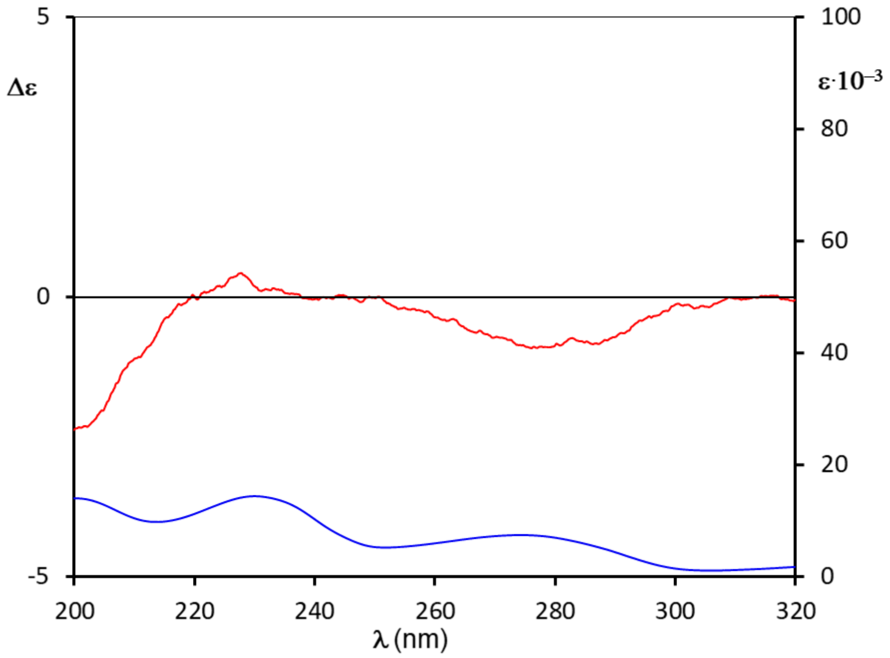

Figure S2. Experimental ECD spectrum for agropyrenol (-)-**2** in CH_3CN.

Table S1 Conformers Boltzmann distribution of **2.**

	DFT/B3LYP/TZVP (Gas Phase)	
Conformers	ΔG (kcal/mol)	% Pop
**a**	0.00	40.3
**b**	0.42	19.8
**c**	0.91	8.7
**d**	1.10	6.3
**e**	1.15	5.8
**f**	1.20	5.3
**g**	1.30	4.5
**h**	1.42	3.7
**i**	1.99	1.4
**j**	2.11	1.1
**k**	2.23	0.9
**l**	2.42	0.7
**m**	2.60	0.5
**n**	2.62	0.5
**o**	2.65	0.5

Table S2. Conformers Boltzmann distribution of (*S*,*S*)-**2a** (*M* and P).

	DFT/B3LYP/TZVP (Gas Phase)	
Conformers	ΔG (kcal/mol)	% Pop
**1-*p***	0.00	14.3
**2-*p***	-0.15	18.5
**3-*p***	0.56	5.5
**4-*p***	-0.61	40.3
**1-*m***	0.60	5.1
**2-*m***	0.74	4.1
**3-*m***	1.33	1.5
**4-*m***	0.17	10.7

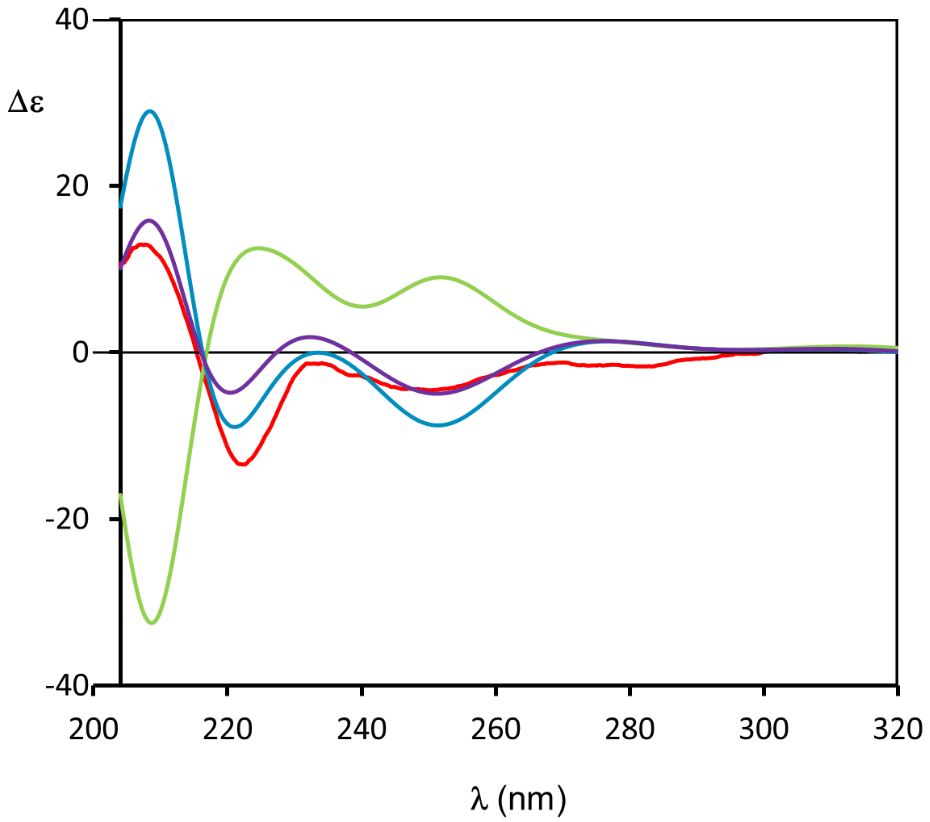

Figure S3. Experimental ECD spectrum of **2a** (red line, THF) and theoretical ECD spectra of (*S*,*S*,*P*)-**2a** (blue line) and (*S*,*S*,*M*)-**2a** (green line) computed at TDDFT/CAM-B3LYP/aug-cc-pVDZ//DFT/B3LYP/TZVP/gas phase and computed spectrum weighed on Boltzmann population of (*S*,*S*,*P*)-**2a** and (*S*,*S*,*M*)-**2a** (violet line). (computed spectra are divided by 5).

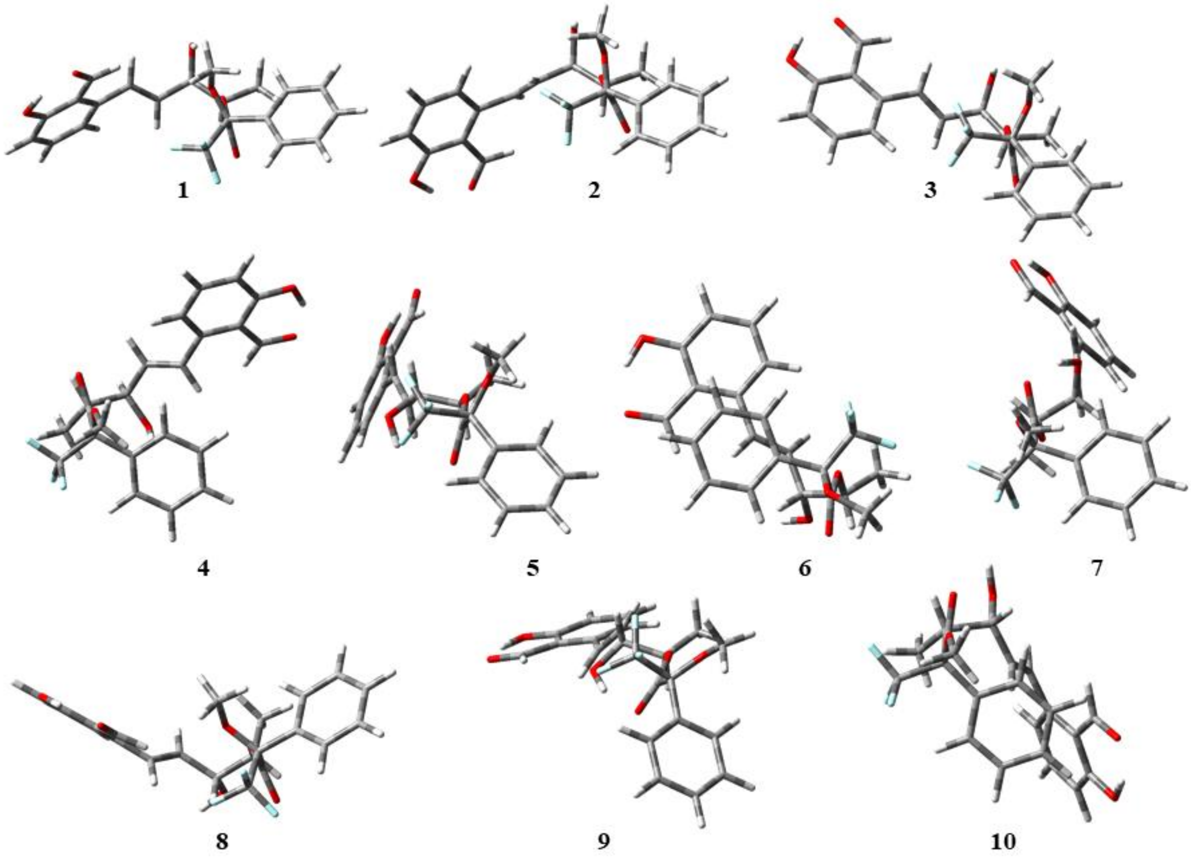

Figure S4. Structures of conformers of 4’-(*R*)-MTPA-**2** computed at DFT/B2LYP/TZVP/ IEFPCM($CHCl_3$) level.

Table S3. Conformers Boltzmann distribution of 4’-(*R*)-MTPA ester of (3’*R*,4’*R*)-**2** computed at DFT/B3LYP/TZVP/IEFPCM($CHCl_3$) level of theory.

		DFT/B3LYP/TZVP (IEFPCM = $CHCl_3$)	
Conformer[a]	rotamer[a]	ΔG (kcal/mol)	% Pop
**1**	d	0.00	40.6
**2**	a	0.52	17.0
**3**	b	0.74	11.6
**4**	d	0.86	9.5
**5**	d	0.89	9.0
**6**	d	1.42	3.7
**7**	d	1.50	3.2
**8**	c	1.53	3.1
**9**	b	1.98	1.4
**10**	d	2.34	0.8

[a]See Figure S4 for conformers structures. [b]See Figure 6 in text for rotamers structures.

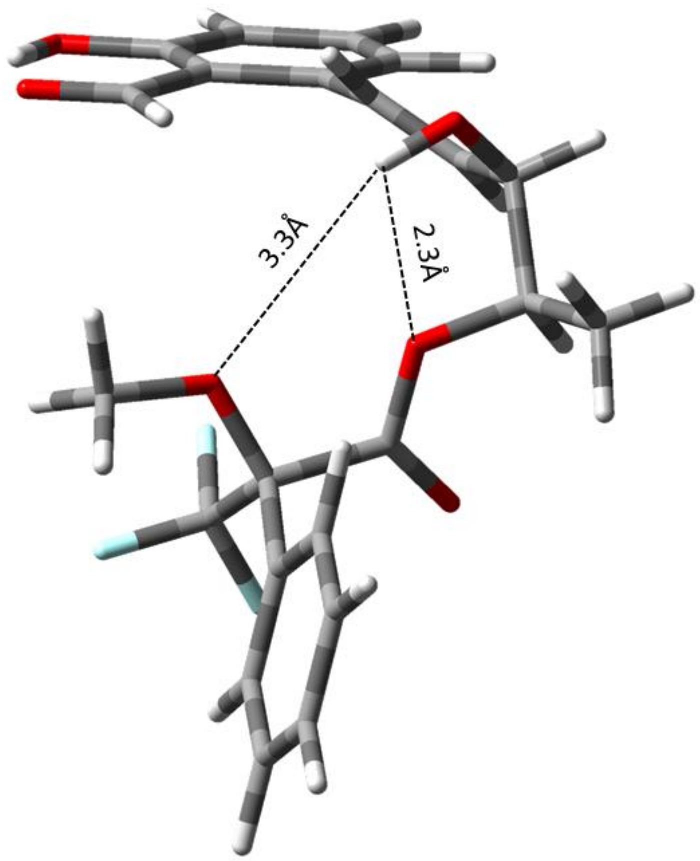

Figure S5. Structure of conformer 1 (rotamer (d)) of (*R*)-MTPA-**2.**

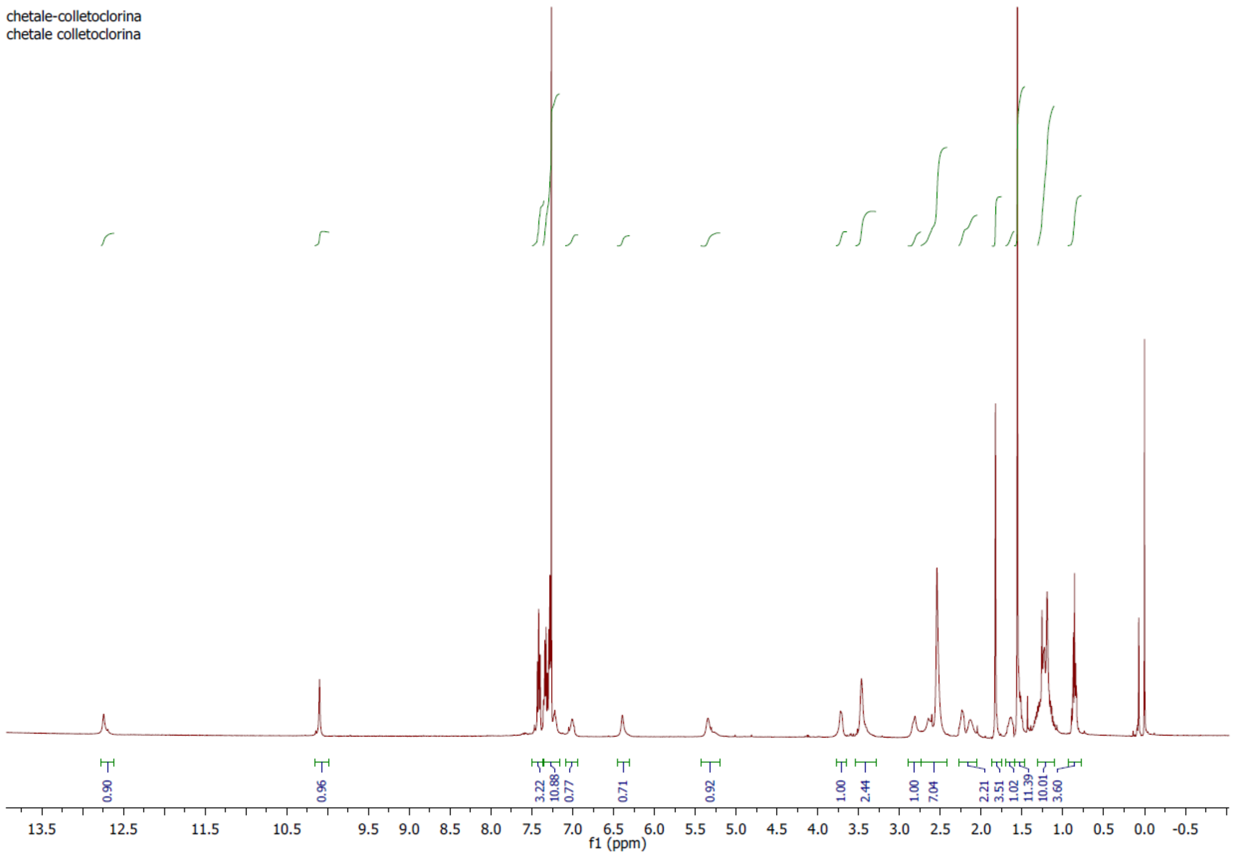

Figure S6. ^{1}HNMR spectrum of **1a**.

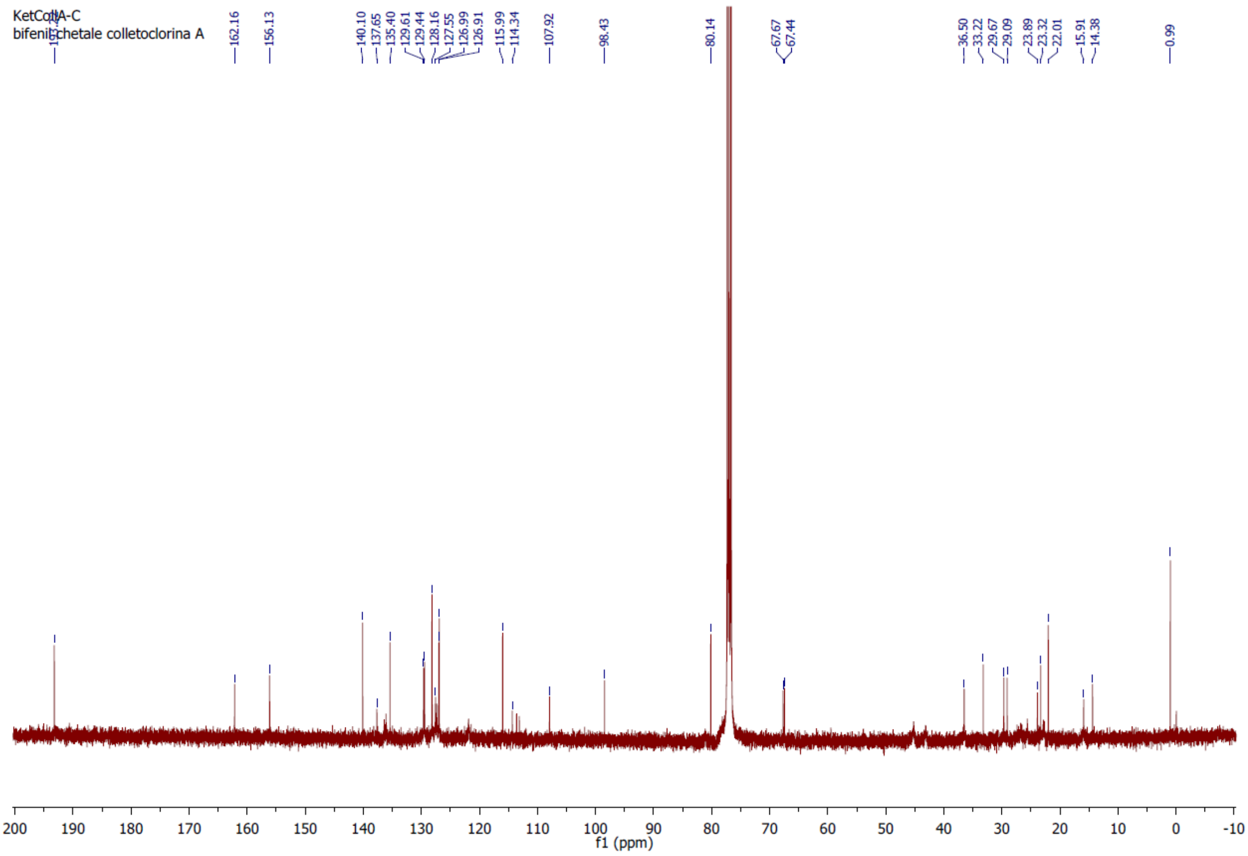

Figure S7. ^{13}CNMR spectrum of **1a**.

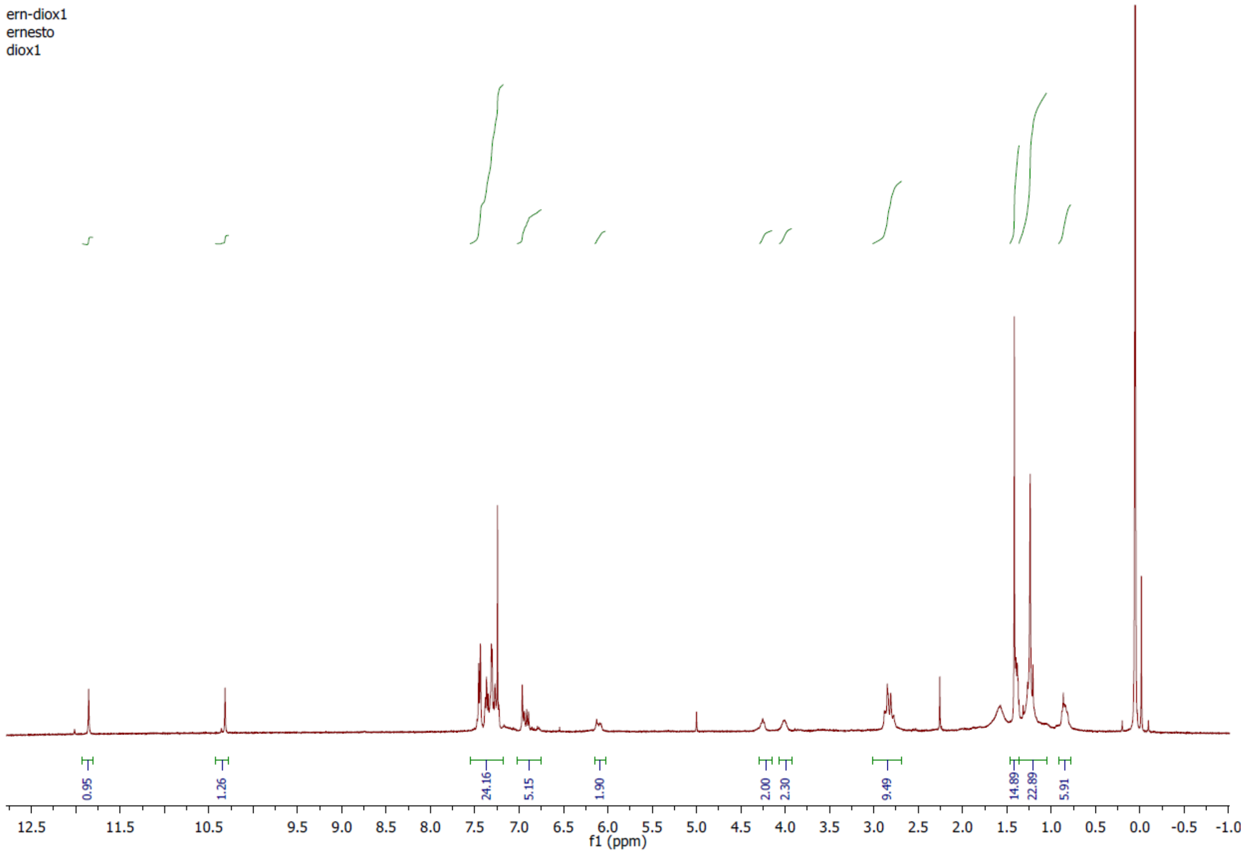

Figure S8. ^{1}HNMR spectrum of **2a**.

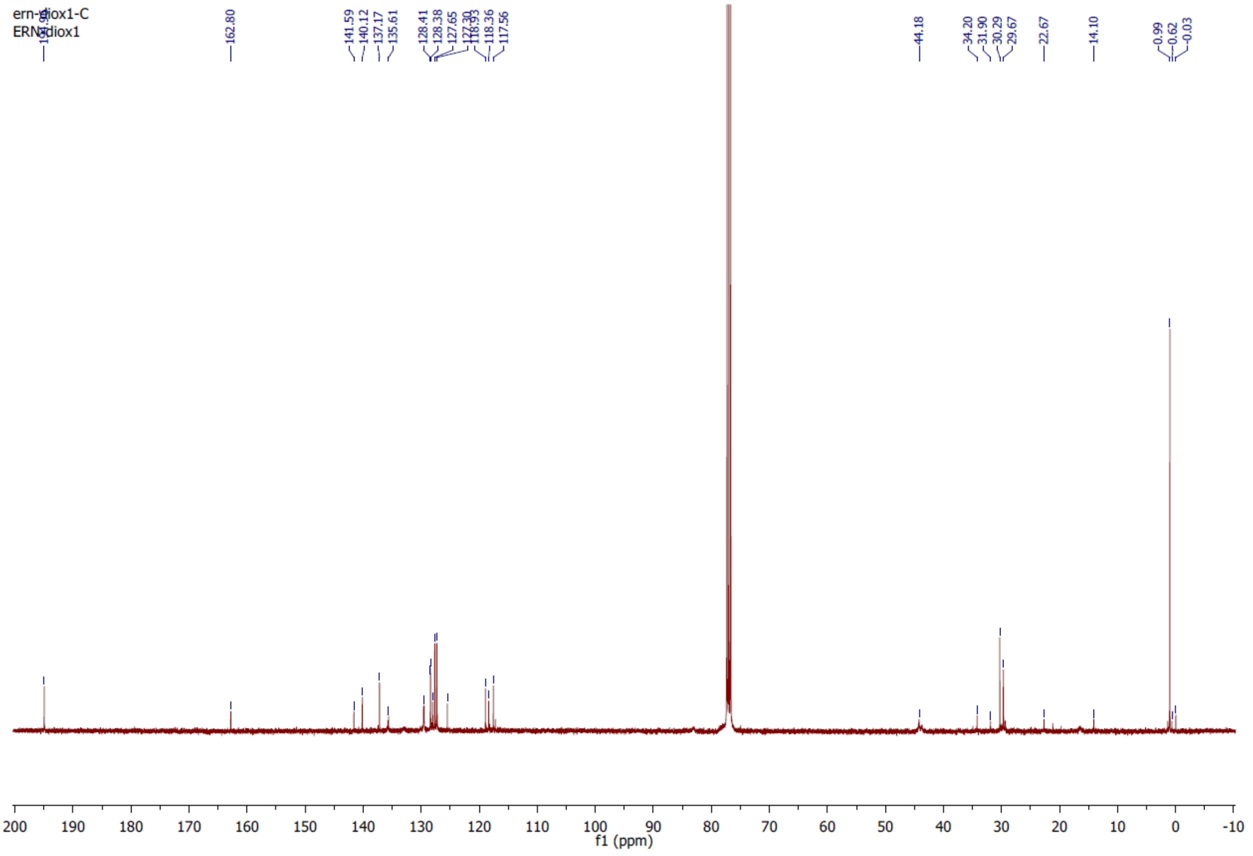

Figure S9. ^{13}HNMR spectrum of **2a**.
